# Polymorphism in the TLR Adaptors of the Toll Signalling Pathway for Use in Livestock Breeding for Health Traits

**DOI:** 10.3390/ijms27104264

**Published:** 2026-05-11

**Authors:** Karel Novák, Kalifa Samaké

**Affiliations:** 1 Department of Genetics and Breeding of Farm Animals, Institute of Animal Science, Přátelství 815, Uhříněves, 104 00 Prague, Czech Republic; 2 Department of Genetics and Microbiology, Charles University, Viničná 7, 128 43 Prague, Czech Republic; kalise.study@gmail.com

**Keywords:** cattle, health traits, immunogenetics, NGS, bioinformatics, genotyping, SNP, functional prediction

## Abstract

Infections in animal production industries can be reduced through targeted breeding of animals with genetically enhanced disease resistance. This type of breeding should be based on a thorough understanding of host defence and its underlying mechanisms. The genes controlling the components of the innate immune system represent a primary target. Their function is highly sensitive to any mutational changes in their structure, which has been optimised through long-term evolution. A source of variability is provided by the polymorphism in the genes encoding components of the Toll signalling pathway as one of the main subsystems of innate immunity. The associated set of genes comprises the group of *TLR* genes encoding the so-called Toll-like receptors (TLRs), as well as genes encoding other key components of the Toll signalling pathway. Specific attention is paid to the genes encoding the crucial interactors of Toll-like receptors, namely the adaptor MyD88 and other adaptors containing the TIR region. Due to the extremely high evolutionary conservation of this region, any structural variation is expected to have functional consequences on the organismal level. The study of the TLR adaptor MyD88, as well as other related adaptors, has been underestimated in farm animal species, as evidenced by the limited number of research outputs. However, this contrasts with the numerous published functional associations for these genes in human medicine. Such a disparity suggests that further research in this direction in farm animal species may yield novel and important findings in the future.

## 1. Introduction

Infections in animal production industries are one of the factors that slow down progress in the sector and negatively impact the economy as a whole. The development of preventive and therapeutic methods requires a thorough understanding of host defence and its underlying mechanisms. In vertebrates, which comprise most livestock species, the immune system maintains the integrity and homeostasis of the organism.

The immune system consists of two subsystems: the innate immune system and the adaptive immune system, which produces antibodies and uses them to target and eliminate specific pathogens. In contrast to this, the innate immune system is primarily composed of innate immune cells, such as neutrophils, natural killer (NK) cells, basophils, eosinophils, mast cells (MCs), innate lymphoid cells (ILCs), mucosal associated invariant T (MAIT) cells, γδT cells, natural killer T (NKT) cells, and other cells. It also includes humoral components such as circulating complement system proteins, cytokines and chemokines secreted by innate immune cells, and various antimicrobial peptides.

The focus on the innate immune system in this review reflects the fact that it is more strongly influenced by inherited genetic variability than the adaptive immune system. This is an obvious fact, although the adaptive system is generally more popular for study.

Although the mammalian immune system comprises both branches, the initial line of defence against infections lies in the innate immune system. Moreover, some components of the innate immune system are required to induce or enhance adaptive immune reactions downstream.

Innate immune processes are characterised by their ability to identify conserved structures of infectious agents. In higher animals, the system is basically formed by a limited number of genes that code for comparatively stable protein products. In contrast, the innate immune system lacks mechanisms analogous to immune gene recombination, such as the V(D)J system, as well as hypermutagenesis taking place in immunoglobulin genes during ontogenesis [1].

The ontogenetically constant structure of the genes involved in innate immunity should not be considered solely as a disadvantage. Their main advantage is their optimised structure, which has been achieved through long-term evolution. The system also benefits from a faster response compared to the adaptive immune system. While the reaction of the Toll signalling pathway in innate immunity requires just minutes to hours, the adaptive response takes days to weeks to be fully expressed [2].

The potential concerning breeding for health traits in livestock using the natural diversity in innate immune genes has been emphasised in reviews by Jungi et al. [3], Novák et al. [4] and Maljković et al. [5]. The present research work is primarily focused on the polymorphism in the genes encoding components of the Toll signalling pathway, one of the main subsystems of innate immunity. It recognises an array of conserved pathogenic determinants and subsequently activates downstream biochemical and cellular responses. The associated set of genes comprises the group of *TLR* genes coding for Toll-like receptors (TLRs) and the genes coding for other key components of the Toll signalling pathway.

In this review, particular attention is given to genes encoding TLR adaptors, namely MyD88, and the genes encoding other TLR interactors, preferably in the innate immune system of cattle as a representative livestock species. Due to the extremely high evolutionary conservation of MyD88, it can be assumed that any variation in its structure should be associated with functional consequences on the organismal level.

The study of MyD88, as well as other related adaptors of Toll-like receptors, has been clearly underestimated in farm animal species, as indicated by the limited number of published studies. This contrasts with the substantial number of functional studies on these genes in human medicine. The current review aims to map this state and specify the opportunities for productive research.

## 2. Toll Pathway in the Frame of Innate Immunity

### 2.1. Toll-like Receptors

As outlined above, the components of the innate immune system mediate rapid recognition of conserved molecules of bacterial, fungal and viral origin. Collectively, these antigenic structures are called pathogen-associated molecular patterns (PAMPs). In parallel, the self-derived molecules released from damaged cells, called damage-associated molecular patterns (DAMPs), can be detected as well. The PAMPs and DAMPs are recognised by the innate immune system upon interaction with the germline-encoded pattern recognition receptors (PRRs), a concept formulated by Charles A. Janeway in 1989 [6]. In the typical case of transmembrane receptor proteins, the hydrophobic sections span the phospholipid bilayer of the plasma membrane, whereas hydrophilic regions stretch out on both the extracellular and intracellular sides of the membrane. A ligand can cause a receptor to change conformation, which allows the receptor to send a signal into a cell. In humans, the transmembrane receptors comprise a large group of 1412 proteins with a multitude of functions in cells and tissues [7].

The PAMPs of microbes are recognised either by the receptors bound to the plasma membrane or by the free cytoplasmic receptors. Based on their localisation, PRRs can be classified into three groups: membrane-bound PRRs, including Toll-like receptors (TLRs) and C-type lectin receptors (CLRs), cytoplasmic receptors, such as nucleotide-binding oligomerisation domain (NOD)-like receptors (NLRs), and RIG-I-like receptors (RLRs) [8].

PRRs activate downstream signalling pathways, and finally, the signal transduction leads to the activation of the terminal transcription factors. The activated transcription factors are then translocated into the nucleus, where they induce the innate immune responses by the production of inflammatory cytokines, type I interferon (IFN), and of other mediators [9].

The cytoplasmic domain of transmembrane receptors serves several purposes. One of its key roles is interacting with proteins, creating a cascade of further signalling events [10]. Toll-like receptors (TLRs) were the first PRRs identified in mammals, and to date, they are the best-characterised ones. The history of TLRs can be tracked back to the studies of the mechanisms by which insects recognise and eliminate infections [11,12]. Subsequently, one of the human homologues of the *Drosophila* Toll protein, TLR4, was shown to drive responses in antigen-presenting cells that induce inflammatory and adaptive immune response [13].

**Figure 1:**
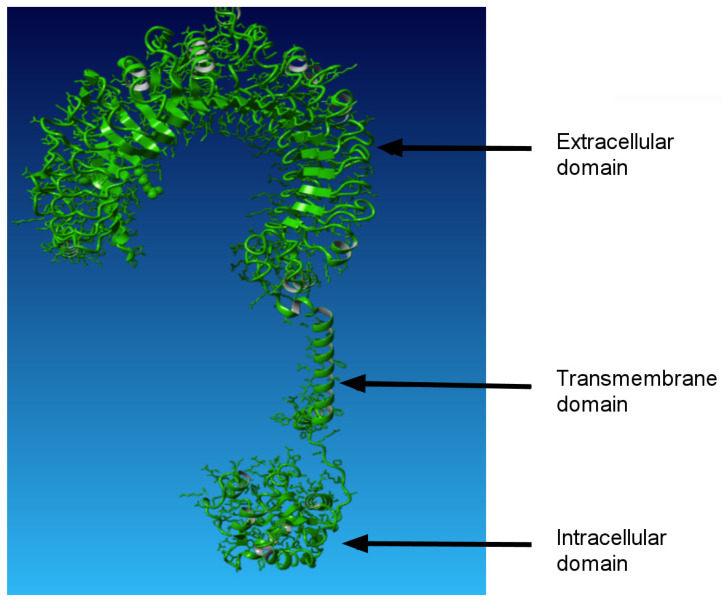
A typical structure formed by transmembrane TLRs consisting of the extracellular, transmembrane and intracellular parts using the example of TLR2 from cattle (for bovine reference sequence UMD3.1.1).

Toll-like receptors are distributed widely across the animal kingdom and are present even in *Porifera* [14]. TLRs are type I transmembrane proteins (Figure 1), consisting of three canonical regions: an extracellular domain with leucine-rich repeats (LRRs), a transmembrane domain, and an intracellular domain tail that contains a conserved TIR region. Its name is derived from “Toll interleukin-1 receptor and resistance” genes. Since the “R” letter in the gene denotation codes refers to plant antimicrobial resistance genes, homologues of the most conserved region of TLRs can also be found in plants [15].

The range of diverse pathogen-associated molecular patterns (PAMPs) recognised by TLRs includes molecules originating from bacteria, fungi, parasites and viruses. These include lipid-based bacterial cell wall components such as lipopolysaccharide (LPS), lipopeptides, and microbial protein components exemplified by bacterial flagellin. In the so-called antiviral receptors TLR3, TLR7, TLR8 and TLR9, the nucleic acids comprising single-stranded or double-stranded RNA and CpG (i.e., 5′-C-phosphate-G-3′) DNA serve as the recognised ligands, as reviewed by Kawai and Akira [16].

In the model vertebrate species, ten human and twelve murine *TLR*s have been characterised. In humans, these comprise series *TLR1*–*TLR10*, while, in mice, they include *TLR1*–*TLR9*, *TLR11*, *TLR12* and *TLR13*, where the homologue of *TLR10* represents a pseudogene [17].

With respect to the function of the product performed, TLR1 and TLR6 recognise bacterial lipoproteins and glycolipids in complexes with the TLR2 molecule. TLR2 itself is essential for the recognition of PAMPs from Gram-positive bacteria, including bacterial lipoproteins, lipomannans and lipoteichoic acids. TLR4 is predominantly activated by bacterial lipopolysaccharides. On the other hand, TLR5 detects bacterial flagellin as the main component of flagella [3]. In the so-called antiviral group, TLR3 is implicated in the perception of virus-derived double-stranded RNA, while TLR9 is required for response to unmethylated CpG DNA. Finally, TLR7 and TLR8 recognise small synthetic interfering (antiviral) RNA molecules. Accordingly, single-stranded RNA was reported to be their natural ligand. TLR11 and -12 (present specifically in mice) have been reported to recognise uropathogenic *E. coli* and a profilin-like protein from *Toxoplasma gondii*, respectively [16,17,18].

In contrast to the plasmalemma localisation of antibacterial TLR series, the receptors of the antiviral group are located in the membranes of the endosomal compartment inside the cell [3]. TLRs are predominantly expressed in tissues involved in immune function, such as the spleen and peripheral blood leukocytes, as well as those exposed to the external environment, including the lungs and gastrointestinal tract. Their expression profiles vary among tissues and cell types [19].

### 2.2. The Mechanism of Toll-like Receptor Action

The first step in TLR signalling is the interaction between TLRs and PAMP ligands. A typical interaction with PAMPs includes the recognition of bacterial LPS by TLR4. Bacterial polysaccharide (LPS), also known as endotoxin, is generally the most efficient immunostimulant recognised by TLR4 among all other components of the bacterial cell walls [20]. In this extremely strong interaction, the lipidic part of LPS, denoted as “lipid A”, is responsible for the most harmful effect associated with Gram-negative (G^−^) bacterial infection—endotoxic shock.

In the acute phase of infection, LPS associated with the LPS binding protein moves in the blood stream. Additional binding takes place to CD14, a glykosyl phosphatidylinositol (GPI) binding protein, expressed on the surface of phagocytic cells. Subsequently, LPS is transferred on the molecule of myeloid differentiation factor 2 (MD-2), which binds to the extracellular part of TLR4 and induces TLR4 oligomerisation. The resulting poly-TLR4 complexes are the key components for LPS signalling.

In the case of TLR2, the activity is dependent on the formation of heterodimers with either TLR1 or TLR6 [21]. This ability results in the formation of specific fixation pockets that facilitate the recognition of triacyl and diacyl lipopeptides, respectively, as released from the bacterial cell walls. The linkage between TLR2 and lipopeptides is strong, although comparatively nonspecific.

Extracellular parts of TLR2, like all other TLRs, are characterised by a varying number of leucine-rich repeats (LRRs) [10]. The interaction with lipopeptides takes place on the level from LRR9 to LRR12: in this place, the hydrophobic residues, fatty acid esters and the internal pocket of TLR2 are located. Recognition of the triacyl lipopeptide by the molecule of TLR2 and heterodimerisation with TLR1 requires the interaction between the C-end of the acyl lipopeptide and hydrophobic channel for phenylbutyrate from TLR1. This match is sufficient to trigger the intracellular signal by the triacyl lipopeptides upon fixation. In the case of TLR6, this hydrophobic channel is blocked by two phenylalanines that prevent the attachment of the third acyl of the lipopeptide [22].

**Figure 2:**
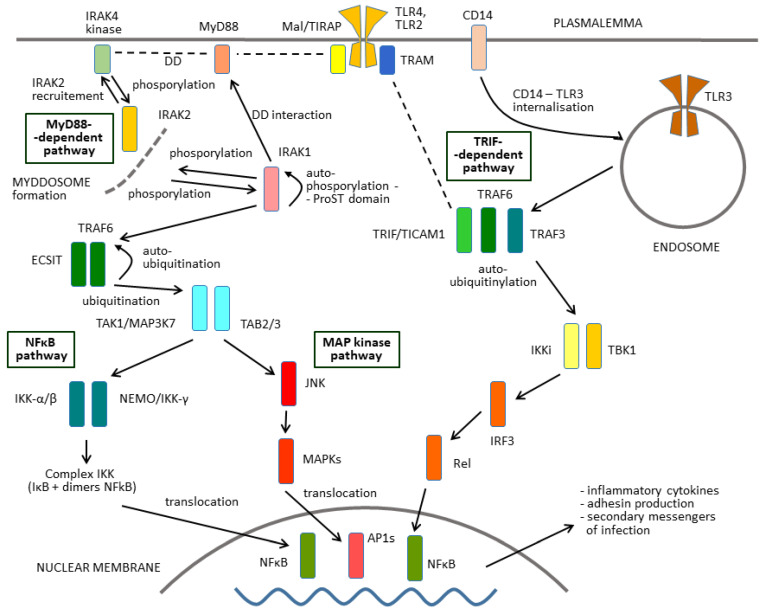
Scheme of the Toll signalling pathway exemplified for TLR4 (antibacterial receptor) and TLR3 (antiviral).

In the next step, the binding of a ligand initiates the recruitment of several further molecules for the receptor complex (Figure 2). Notably, these components include adaptor molecules characterised by the presence of the TIR domain, like the TLR molecules themselves, and by the so-called death domain (DD) in the myddosome complex formation. First, they comprise the product of the myeloid differentiation primary response gene 88 (MyD88). This group of adaptors also include TIR domain-containing adaptor Mal/TIRAP (MyD88 adaptor-like), TIR domain-containing adaptor-inducing interferon β (TICAM1/TRIF) and TRIF-related adaptor molecule (TRAM).

### 2.3. Downstream Signal Transfer

Upon activation of a TLR molecule by the interaction with a ligand, the kinase activity of the molecules downstream of TLR is increased. The line for signal transfer to the terminal nuclear factor-κB (NF-κB), which functions as a transcription factor, consists of a set of positive and negative regulatory elements (Figure 2). Every kinase in the signalling pathways, in its turn, phosphorylates a succeeding adaptor or another protein. The addition of a phosphate group will alter the structure of the next protein in the sequence, turning it from an inactive kinase to an active one. The ensuing phosphorylation cascades will eventually cause the cell to behave differently, either by changing the expression of genes or by changing the activity of proteins that are already present in the cytoplasm.

However, upon recognition of their agonists, TLRs activate not just a single pathway but two parallel signalling and interacting pathways (Figure 2). The first Toll signalling pathway is MyD88-dependent, whereas the second one requires the presence of TRIF.

Activation of the MyD88-dependent pathway (left side of Figure 2) includes protein kinases IRAK1, -2, -3, and -4 in the first stage. The next key member of this pathway is transforming growth factor-beta-activated kinase 1 (TAK1, also MAP3K7). Although the mechanisms of TAK1 activation within this complex remain unclear, K63-linked ubiquitination or close proximity-dependent transphosphorylation may be responsible for TAK1 activation [23].

**Figure 3:**
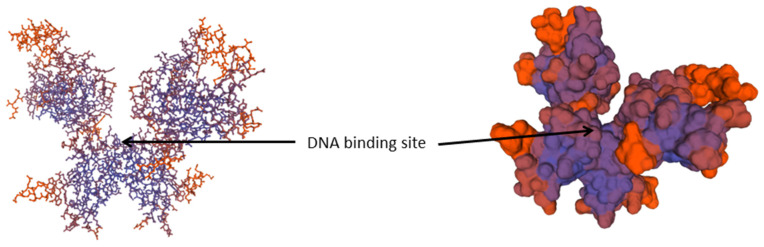
The conformation of the central transcription factor NF-κB (for bovine reference sequence UMD3.1.1).

TAK1, in turn, activates two parallel branches of the pathway downstream: the NF-κB pathway and MAPK pathway. The left branch leads to activation of the IKK complex (IκB) and, subsequently, to NF-κB activation. The role of the IKK complex is essential here [24]. The complex is composed of the catalytic subunits IKKα and IKKβ and the regulatory subunit NEMO (NF-κB essential modulator, also called IKKγ). The activation of IKK includes phosphorylation, ubiquitinylation and degradation of the IKK proteins (Figure 2). The upstream TAK1 binds to the IKK complex through ubiquitin chains, which allows it to phosphorylate and activate IKKβ [25]. The IKK complex in turn phosphorylates the NF-κB inhibitory protein IκBα, which subsequently undergoes proteasome degradation, allowing NF-κB to translocate into the nucleus. NF-κB then can induce proinflammatory gene expression, including TNF, IL-1β, and IL-6 [26]. The NF-κB structure is demonstrated in Figure 3.

In parallel, TAK1 activates MAPK family members in the sub-pathway. This family includes ERK1/2, p38 and JNK, which mediates activation of AP-1.

A hallmark of MyD88-independent signalling is induction of the dendritic cell maturation pathway and induction of type 1 interferon (IFN-β) [9].

On the other hand, a major parallel signalling pathway from the TLR–ligand complex (right side of Figure 2) leads to TRAF6 (tumour necrosis factor (TNF) receptor-associated factor 6) and TBK1 (TANK-binding kinase 1) [23]. This results in the activation of the P50/c-rel heterodimer from nuclear factor-κB [16]. The released NF-κB dimers are then activated via posttranslational modifications and translocated to the nucleus, where they bind to specific sequences and induce the transcription of target genes.

As can be seen in Figure 3, NF-κB proteins are transcription activators that are present in the cell in the form of dimers. It should be noted that both proteins NF-κB1 and NF-κB2 are present in a long and a short form. The short form can be released during the translation process. The two forms are denoted as p50 and p105 in NF-κB1 and p52 and p100 in the case of NF-κB2. While the heavier proteins perform the role of specific inhibitors of transcription, the lighter proteins join with the protein c-Rel or with other Rel proteins and form transcription factors. Five members of this protein family are known in mammals, namely p50, p52, c-rel, p65 (also denoted as relA) and relB. The complexes of transactivators, which are the most abundant, are heterodimers p50/p65 (that correspond to the originally described activity of NF-κB), but other combinations also occur frequently. Subunits p50 and p152 are synthesised in the form of cytoplasmic precursors, namely p105 and p100.

As described above, we can reconstruct the full pathway from the Toll-like receptors to the terminal transcription factors and, moreover, in different cell types. However, it should be taken into account that TLRs are expressed on a variety of immune cells, including macrophages, dendritic cells (DCs), B cells, specific types of T cells, and even nonimmune cells such as fibroblasts and epithelial cells. This can be also associated with a variation in the pathways.

For almost two decades, NF-κB as the terminal member of the Toll signalling pathway has been used as a standard example of an inducible transcription factor. NF-κB is still the focus of extensive research in view of a wide range of triggers that activate it and the vast number of genes that it regulates [27]. According to updated data from the specialised server run by the Thomas Gilmore lab in Boston University (https://www.bu.edu/nf-kb/gene-resources/target-genes/, accessed on 16 November 2025), the activity in this transcription factor affects over 550 genes. Consequently, NF-κB can be considered a major pleiotropic factor for many phenotypic traits. To date, over 80 related traits on the organismal level have been reported in biomedicine. However, the basic function of the NF-κB signalling pathway is to promote inflammatory immunological responses.

## 3. Regulation of TLRs by Co-Receptors and the Role of MyD88

TLR expression is not stationary but is rather modulated in the course of response to pathogens by various cytokines and environmental factors. The signalling pathway itself is affected by a number of coreceptors and adaptors. They encompass proteins that closely interact with the main members of the pathway via the TIR and DD conserved domains.

**Figure 4:**
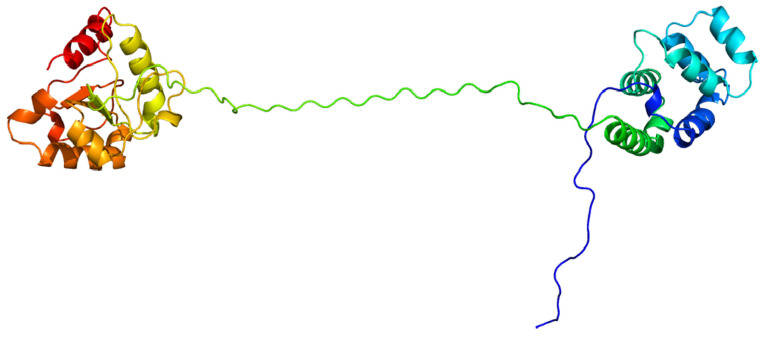
Structure of bovine MyD88, the main adaptor of Toll-like receptors with structures of the TIR domain and death domain; modelled using Phyre 2 [30] (for bovine reference sequence UMD3.1.1). The blue colour corresponds to the N-terminus of the protein.

It is significant that the TIR domain of TLRs mediates interactions with the TIR domain in the adaptor molecule MyD88, which was described by Medzhitov et al. in 1998 [28]. While MyD88 is the abbreviation for “myeloid differentiation primary response 88”, the corresponding gene is denoted as *MYD88*. The encoded protein consists of an N-terminal death domain and a C-terminal Toll-interleukin-1 receptor domain (Figure 4). This molecule is used as a key adaptor by all TLRs except for TLR3, which uses the adaptor molecule TRIF instead for downstream signal transmission [29].

The process of regulation can be specified for individual steps of the pathway (Figure 2). After TLR engagement, MyD88 forms a complex with IRAK (Il-1R-associated kinase) family members [31]. The resulting complex is referred to as the myddosome [32]. Interactions with members of the IRAK group are enabled simply by binding of MyD88 to the proximal part of the TIR domain of activated TLRs and via the DD domain.

Subsequently, IRAK4 kinase activates IRAK1, which is then autophosphorylated at several sites [33]. The phosphorylated IRAK1 is then released from MyD88.

This chain of events leads to the activation of the transcription factor NF-κB as the final member of the pathway via the kinase complex IκBα + IKKβ + IKKγ; the components are also referred to as IKK1, IKK2 and NEMO. This complex is the key regulator of the entire pathway [24]. A hallmark of MyD88-independent signalling is induction of the dendritic cell maturation pathway and induction of type 1 interferon (IFN-β) [9].

Analogically to the MyD88 pathway, the TRIF-dependent parallel signalling pathway is activated as well [34]. Similarly, TNF (“tumour necrosis factor”) receptor 6 (TRAF6) is activated by its recruitment to activated receptors, TLRs (and also to IL-17R, IL-25R, and CD40), and by subsequent auto-ubiquitination.

In addition, several other transmembrane molecules that modulate the TLR signalling pathways have been identified. CD14, a glycophosphatidylinositol-anchored transmembrane protein, performs as a co-receptor of TLR4 and MD-2 in LPS recognition. CD14 induces endocytosis to promote TLR4 internalisation into endosomes. This process is mediated by ITAM and dependent on SYK and PLCγ2. CD14 has also been demonstrated to be required for TLR7- and TLR9-dependent induction of proinflammatory cytokines [35].

As emphasised above, additional adaptors containing the TIR domain are crucial for signal transduction along the Toll signalling pathway. For example, the TLRs bound to the adaptor MyD88 additionally tightly interact with the adaptor Mal/TIRAP [36,37]. This protein is structurally related to MyD88 and was discovered on the basis of its ability to mediate TLR4 signalling. Primarily, Mal/TIRAP was demonstrated to affect LPS-induced IFN-β production in vitro. A subsequent study in a mice line with the inactivated Mal gene demonstrated that Mal is involved in MyD88-dependent NF-κB activation at the stage after LPS stimulation and ligand-dependent engagement of TLR2 [38].

The formation of the complex of TLR/MyD88/Mal is mediated simply by the interaction of the corresponding TIR and DD regions. As a result, if we consider this extension of functional dimers TLR1/TLR2 and TLR2/TLR6, the resulting active complexes will contain from three to five different TIR regions belonging to different molecules, namely TLRs, IRAK1, IRAK6, and Mal.

On the other hand, some factors act in the opposite way as inhibitors of the TLR system. IRF-7 (interferon regulatory factor 7) is the regulator of transcription, which is necessary for gene expression in type I interferon-α (IFN-α). The IRF-7 gene is expressed mostly in lymphoid cells, and the transcription of IRF-7 is caused by IFN and postranslationally activated by phosporylation of serine residues on the H-end. Some of these residues are conserved and identical to IRF-3. IKKε and TBK-1 (TANK-binding kinase) are key regulators of IRF-3 and IRF-7 during the pathway activation in those cells that were exposed to viruses or activated by free dsRNA via TLR3, IKKε and TBK1 [36].

The components of the TRIF-dependent signalling pathway are also necessary for activation of IRF-3. The role of IRF3 in the LPS-induced gene expression of IFN-β and during endotoxin shock has been confirmed in a study in a mouse line with a knockout gene [39]. Nevertheless, the molecular mechanisms that regulate the response to LPS independently of MyD88 via the activation of IRF-3 and, subsequently, NF-κB are still not known sufficiently.

## 4. Antimicrobial Action Ascribed to the Toll Signalling Pathway and Other Phenotypic Effects

### 4.1. Known Examples from Model Species

The activation of MyD88-dependent and TRIF-dependent signalling pathways, both initiated at TLRs, leads to the secretion of inflammatory cytokines, interferons of type I (IFN), chemokines and antimycobacterial peptides. Both pathways also cause the activation of neutrophils and macrophages and induction of IFN-stimulated genes. This results in the killing of infectious microorganisms and the activation of adaptive immunity [40].

MyD88^−/−^ mice infected with *M. tuberculosis* display reduced expression of gamma interferon (IFN-), tumour necrosis factor alpha (TNF-), and nitric oxide synthase (NOS). This observation has suggested that MyD88 controls the infection by regulating the production of these mediators [41]. The above-described studies and the high genetic similarity (99.95% identity at the nucleotide level) of the *M. tuberculosis* and *M. bovis* genomes [42] collectively provided biological plausibility to the hypothesis of a functional role of the MyD88 gene against bovine tuberculosis infection.

### 4.2. Examples from Human Medicine

Generally, the signalling pathways leading to NF-κB play an important role in immune inflammatory responses in human medicine.

Consistently, many immunotherapies are based on targeting the PRRs. For example, the pattern recognition receptor Nod2 (nucleotide-binding oligomerisation domain-containing protein 2, also CARD15 or IBD1) has been associated with the development of Crohn’s disease [43]. In addition, the TLR agonist molecules are standardly used in vaccine development as adjuvants or immune stimulants.

It has been shown recently that this pathway is also involved in antiapoptotic responses, both in the course of normal differentiation (especially in the haematopoietic system) and in carcinogenesis.

Innate and adaptive immune responses to mycobacteria rely on Toll-like receptors (TLRs), which sense several mycobacterial components. Sensing of mycobacterial DNA requires TLR9, while heat shock protein 65 (HSP65) requires TLR4 and lipomannan (LM), lipoarabinomannan (LAM), 19-kDa lipoprotein (19LP), and soluble tuberculosis factor (STF) require TLR2 [44].

This knowledge of the response to human mycobacterial pathogens is obviously transferable to the mycobacterial pathogenesis in livestock species.

### 4.3. Known Examples from Farm Species

Additionally, in livestock species, TLRs have been, in many cases, demonstrated to be the main receptors for important pathogens. These experimental findings are often just the extension of results obtained from human or in laboratory models like mice.

This rule has been confirmed for the recognition of mycobacterial pathogens. Pathogenic mycobacteria are intracellular bacteria that survive inside the host macrophages thanks to a number of complicated mechanisms. Pure components of the mycobacterial cell wall preferentially activate TLR2 and, to a lesser extent, TLR4 [18].

It is assumed that TLR4 is involved in the complex of bovine infectious respiratory diseases associated with *Mannheimia* (*Pasteurella*) *haemolytica* [44]. *TLR2* and *TLR4* genes are also highly expressed during mastitis caused by *Streptococcus aureus.* Since TLR4 is involved in PAM recognition, mutation in the receptor can restrict the host response to certain pathogens. For this reason, TLR4 is highly polymorphic, and its enhanced expression is associated with intramammary infections—IMIs. This gene can be a potential candidate for exploitation in marker-assisted selection for increased resistance to mastitis in milking cattle [45]. Some single-nucleotide polymorphisms (SNPs) in *TLR2* have been reported to affect mastitis prevalence in Holstein, Simmental and Sanhe cattle [46], while some other SNPs are associated with paratuberculosis susceptibility [40,47].

Mutations in the *TLR1* and *TLR4* genes investigated in the pioneering work by Mucha et al. in 2009 [48] caused a weaker immune response to lipopeptides, lipopolysaccharides, components of the microbial cell wall and increased susceptibility to invasive aspergillosis (i.e., mycotic pneumonia). Several characteristic fungal PAMPs that are localised in the cell wall or on a cell surface of fungi can be recognised by TLR2 or TLR4 as well [49].

Components of protozoal parasites are also detected by TLRs. TLRs are activated by *Trypanosoma cruzi*, *Trypanosoma brucei*, *Toxoplasma gondii*, *Leishmania major* and *Plasmodium falciparum*. The corresponding ligands include glycosyl phosphatidyl pirositol-mucin (tGPI-mucin) and glycoinositol phospholipids (GIPLs).

The testing of artificial ligands to TLR4 and of purified LPS in the study by Conejeros et al. [50] described a fast change in the size and morphology of bovine polymorphonuclear neutrophils (PMNs) as fast as 10 s after the beginning of stimulation. In addition, studies in human medicine confirm the assumption that TLR ligands can induce direct activation of PMNs and consequently enhance the ability to destroy bacteria.

Last, but not least, there is the line of research represented by Japanese quail, which is both a domestic livestock species and, in parallel, a model species for basic research [51].

In addition to their function in innate immunity in vertebrates, the pleiotropic effects of Toll-like receptor coding genes (*TLR*s) have been demonstrated in many other traits, both on the molecular and organismal levels [52]. For example, effects on parturition in a mouse model have been demonstrated using a murine line with a *TLR4* gene knockout [53].

## 5. Natural and Generated Diversity of TLRs and the Documented Consequences

### 5.1. General Toll Signalling Pathway Polymorphism

Upon the identification of a homologue of the particular *TLR* in the given species, a wide-scale screening for diversity usually succeeds. The current state of sequencing technologies implies a wide use of parallel sequencing. The next step is usually aimed at the identification of functional consequences, either by in silico modelling or in association studies on the organism level.

The variants occurring in individual animals are statistically correlated with the effects of the traits of interest measurable on the organismal level [54]. These types of studies provide a link between the effects postulated at the molecular level using prediction programmes and the final effect, which is observable after many basically unknown processes and interactions on the organismal level, leading to the final phenotype. This allows bridging the gap in knowledge between the description of the variant on the molecular level and the available evaluation of the resulting traits.

Known traditional examples of differential susceptibility to infections that might be linked to TLR pathway variability comprise a range of cases.

### 5.2. Known Mutations in the TLRs and Their Consequences in Livestock Species

Spontaneously occurring differential susceptibility to infections has been described in a series of livestock species [55]. In cattle, the known effects of the genetic predisposition to udder bacterial infections (*E*. *coli*-caused mastitis) are generally known and have been thoroughly discussed by Detilleux [56]. Consequently, in livestock species, *TLR*s have been considered prospective targets for breeding, primarily as the representatives of innate immunity genes [57].

On the other hand, natural variations in bovine candidate *TLR* genes have been repeatedly reported for a panel of world cattle breeds [58,59,60] and are documented in public nucleotide sequence databases. Some of the variants in coding regions are predicted to disturb the function of the protein product. As expected, associations with health traits in cattle populations have been reported for naturally occurring variants [61,62,63]. For instance, the predicted functional change of c.2021C>T in *TLR4*, affecting the transmembrane region, causes a shift in somatic cell count in milk [45,64]. In *TLR2*, the 1047G>T and 1313G>A nonsynonymous polymorphisms have been reported to be effective in increasing susceptibility to paratuberculosis (PTB) [40,47,61].

The variability in innate immune genes in livestock species, namely in cattle, has become the subject of many published association studies [3,4,5]. For Toll signalling genes in cattle, this approach has been developed in several leading laboratories. The first disease evaluated for association with the *TLR* polymorphism was mastitis as a continually monitored infection in cattle [65]. The work indicated the association of *TLR2* and *TLR4* polymorphisms with clinical mastitis in Norwegian Red cattle. The finding of the effect of the *TLR4* variants has been independently confirmed for the Canadian Holstein population [45].

Another model system is the susceptibility to paratuberculosis infection (caused by *Mycobacterium avium* subspecies *paratuberculosis*). One of the earliest studies in this area, conducted at the University of Veterinary Medicine in Košice, examined the effects of polymorphism in bovine *TLR1*, *TLR2* and *TLR4* genes [48]. Consistently, the effect of single-nucleotide polymorphisms in *TLR2* on mycobacterial infection in cattle has been revealed [61,66].

In cattle, the products of the *TLR1*, -*2*, -*4*, and -*6* genes recognise polysaccharide or glycoprotein ligands released from the cell walls of Gram-negative and Gram-positive bacteria, while the *TLR5* product recognises the flagellin protein from bacterial flagella [3,67]. On the organismal level, their role has also been demonstrated in many immune and nonimmune traits [52]. For instance, the predicted functional change encoded by c.2021C>T affecting the transmembrane region of the *TLR4* product was associated with a shift in somatic cell count in milk as an indicator of inflammation [45,64].

Nevertheless, in many cases, synonymous mutations without a change to the protein structure or even mutations in the noncoding regions of bovine *TLR* genes have demonstrated associations with phenotypic traits [68]. For example, noncoding 1313G>A in *TLR2* increases susceptibility to PTB [62].

On the other hand, it can hardly be surprising that the variability in the TIR regions of TLRs is strictly limited [69]. It can be assumed that any new mutation disturbs the interaction with two other members of the receptor complex with adaptors and negatively affects the function of it. If this effect is not compensated for by complementary mutations in other members of the complex, any new mutation is negatively selected and sooner or later lost from the population. This assumption is confirmed by the very low variability of TIR regions observed in population studies in the main model animal species.

On a wide scale, associations of the diversity in the full series of bovine *TLR* genes with paratuberculosis have been documented by Fisher et al. [63]. This approach has been extended to SNPs organised in haplotypes in the same model of cattle vs. *Mycobacterium* by Juste et al. [70]. This observation found confirmation in many subsequent works performed in different genetic backgrounds of other cattle breeds and, moreover, kept under different climatic and epidemiologic conditions, e.g., [55]. For *TLR1* and *TLR9*, the effect of variants on mycobacterial infection has been reported for the Holstein cattle population in China [71].

## 6. Natural Variability of TLR Adaptors Including MYD88

### 6.1. Known Mutations in the TLR Adaptors and Their Consequences in Model Species

Experiments performed on laboratory model species like mice are essential for the extending research in medicinal research and in livestock species. Although most conclusions on the role of variability in the TLR adaptors are still based on the study of the human innate immune genes, sooner or later, they find confirmation in model species in a narrow sense, such as mice, hamster or rats.

The main advantage of using mice in this area of research is represented by mouse lines with a deleted inactivated MYD88 gene. The Myd88^−/−^ or Myd88^+/−^ animals allow for a direct proof of the role of MyD88 in the studied phenomenon, including infectious diseases. Since these lines are unresponsive to bacterial endotoxin (LPS of G^−^ bacteria), the role of MyD88 is clearly delimited [72].

In this way, the role of MyD88 in resistance to the causative agent of Q fever, which is intracellular bacterium *Coxiella burnetii*, has been demonstrated [73]. This intracellular bacterium is the pathogenic agent of the zoonotic disease Q fever, which usually manifests as a respiratory infection following inhalation of aerosolised bacteria shed by infected small ruminants. The persistence of the *C. burnetii* in the cells of the Myd88^−/−^ mouse line provides the basic evidence of the role of innate immune signalling in resistance formation in this case.

Similarly, Myd88-deficient mice have been shown to be susceptible to *Leishmania* infection [74]. The use of MyD88-deficient mice line also allowed to uncouple the innate and acquired immune responses against *Mycobacterium tuberculosis* [75]. It has also been shown that Myd88-deficient macrophages did not produce TNF and NO upon mycobacterial stimulation. Nevertheless, the adaptive immune response was not sufficient to prevent the infection development by itself.

Altogether, Myd88-deficient mice have been shown to be susceptible to 46 pathogens. This group included 27 bacteria, eight viruses, seven parasites, and four fungi [72].

Since the Toll signalling pathway ending with NFKB1 is highly pleiotropic, the effect of functional SNPs in the murine MYD88 of rats can be observed in such an atypical trait like decompression sickness, along with mutations in NFKB1 itself [76]. The autoimmune response is supposed to be involved in this case.

### 6.2. Known Mutations in TLR Adaptors in Human Medicine

The TLR- and IL-1R-dependent immune pathways in mice, which are mediated by MyD88 and IRAK-4, seem to be sufficient to combat a wide array of pathogens at most ages. In contrast, human TLR- and IL-1R-dependent immunity, also mediated by MyD88 and IRAK-4, seems to be fully effective only against some bacteria. Moreover, this system is more efficient in infancy and early childhood [72].

The importance of the conserved TIR domain in the Toll receptor adaptors has been documented by George et al. in 2011 [77]. Two variants of MyD88 obtained in a human cell line, S34Y and R98C, interfered with the myddosome assembly, namely with the acquisition of MyD88 and IRAK4 molecules. In parallel, this observation on the mutational effects of human IRAK4 and MyD88 genes has been confirmed by Yamamoto et al. [78]. Nagpal et al. [79] complemented this finding by induced changes to the TIR domain of Mal/TIRAP in a human cell line that resulted in the loss of MyD88 binding and in reduced signalling by the TLR2/TLR4 dimer. As might be expected, patients with defects in MYD88 have an increased susceptibility to pyogenic bacterial infections [80].

Effects of mutations in human MyD88 on the course of bacterial infections, namely in the tuberculosis model caused by *Mycobacterium*, has also been demonstrated for the 938C>A polymorphism that is associated with disease susceptibility to mycobacterial infection [81]. The same association can be observed in the rs6853 variant of human MyD88 [82]. Moreover, the same effect was exerted by the mutation rs8177374 in the Mal/TIRAP molecule, which functions as a MyD88 interactor.

MyD88 polymorphism also controls acquisition and transmission of *Borrelia burgdorferi*, a bacterium transmitted to animal and human hosts by *Ixodes scapularis* ticks [83]. MyD88 is also required for the efficient control of Q fever in humans, which is a typical zoonosis caused by the G^−^ bacterium *Coxiella burnetii* [73]. This pathogen is located intracellularly in phagolysosomes and transferred to human patients from sheep, horses, cattle, goats and pigs as hosts, often as an occupational disease. Similar to the pathogenic strains of *Borrelia*, the majority of *Coxiella* species are harboured by ticks as endosymbionts. However, it seems that these forms of *Coxiella* do not possess virulence genes for higher animals. The role of the MyD88 adaptor was demonstrated using a MyD88-deficient mice line, as discussed above [73].

In light of its documented role in *Borrelia* and *Coxiella* infections, it is not surprising that MyD88, together with NOX2 (NADPH oxidase 2), is involved in the regulation of another bacterial infection, pulmonary granuloma, caused by the G^−^ bacterium *Propionibacterium acnes* [84]. The MyD88-deficient mice line was used to obtain evidence in this case as well.

In *Streptococcus pneumoniae* (*Pneumococcus*), as another model bacterium causing pulmonary infections, this time G^+^, the effect of the polymorphism in MyD88 on the severity of infections has been demonstrated in an association study. Two other interactors on the TIR region level, namely IRAK4 and IKKγ (inhibitor of NF-κB kinase regulatory subunit γ-NEMO and coded by the IKBKG gene), are involved in *S. pneumoniae* susceptibility [85]. This observation has been independently confirmed by Carrasco-Colom et al. [86] for polymorphisms in MyD88 and in the TLR interactors IRAK1 and IRAK4 in the case of severe pneumococcal disease.

The polymorphism of MyD88 and other TIR-containing adaptors has been shown to also play a role in protozoan diseases. The importance of MyD88 in the control of protozoan infections is underscored by the association between the occurrence of ocular toxoplasmosis, caused by a protozoan *Toxoplasma gondii*, and polymorphism in the gene *MYD88*, as demonstrated by Aloise et al. [87]. Curiously, this association has been extended to the polymorphism in the DNA repair enzyme APEX1 (apurinic/apyrimidinic endonuclease 1).

Similarly, a role of polymorphism in Mal as another TIR-containing adaptor in the severity and outcome of malaria as caused by *Plasmodium falciparum* has been reported by Ammar et al. [88], namely, the effect of rs8177374 polymorphism has been detected. This observation has been independently confirmed by Rani et al. [89] for the same polymorphism in Mal in relation to the level of malaria manifestation upon *P. falciparum* and *P. vivax* infections. The effect of the Mal structure might have been mediated by cytokines IFN-γ, TNF-α, IL-10, and TGF-β in this case.

The role of MyD88 in signalling against viral infections is documented by the demonstrated involvement of this adaptor in signalling for the response to *Rhinovirus* infection [90].

In the case of protozoa, MyD88 has also been demonstrated to participate in immune responses. Along with TLR9, it controls the resistance towards *Leishmania guyanensis* infections [91], as supported again by the observation in the MyD88^−/−^ mouse line.

The polymorphism in MyD88 is also known for its role in different pathological states, even those not directly caused by infections. For example, the participation of MyD88 is supported by the induction of the profibrotic environment in chronic hepatitis via the MyD88-dependent pathway [92].

Polymorphisms in MyD88 are also known to have direct effects on immune traits in general. Therefore, it is not surprising that the effect of polymorphism in the members of the Myd88 pathway, including MyD88 itself, can be observed directly on the organismal level as diseases in the category of immunological disorders. The role of MyD88 polymorphism and in the functionally related signalling molecules in the formation of systemic lupus erythematosus has been reported by Wen et al. [93]. Similarly, a single-nucleotide polymorphism in the 3′-untranslated region of the MyD88 gene has been demonstrated to affect the incidence of Buerger disease (thromboangiitis obliterans), i.e., an inflammatory condition causing blood vessels in the hands and feet to swell and form clots [94].

Similarly, variation in the Mal protein, another TIR-containing adaptor, exerts broad effects on immune function. An et al. [95] demonstrated that genetic variants in the Mal protein are associated with atopic dermatitis, and Holtick et al. [96] raised the role of the Mal variant with Leu 180 in protection against graft-versus-host disease.

A more critical role of human MyD88 polymorphism, leading to septic shock in surgical patients, has been reported by Jiménez-Sousa et al. [97].

Finally, the mutations in human MyD88 are considered to be an important factor allowing for development of lymphomas. Recurrent mutations in MYD88 and TBL1XR1 (transducin beta-like 1 receptor 1) are supposed to be factors contributing to lymphomas of the central nervous system [98]. Consistently, the L265P mutation in Myd88 is considered a prognostic factor for patients who are treated for diffuse large B-cell non-Hodgkin lymphoma [99]. An analogical role of MYD88 mutations has been reported for patients with chronic lymphocytic leukaemia by Maleki et al. [100]. In the case of intraocular lymphoma, the L265P mutation in MYD88 serves as a diagnostic marker [101].

Polymorphism in human MyD88 and Mal/TIRAP is also involved in tumorigenesis in solid tissues, including gastric tumorigenesis, which is known to be mediated in part by TLR2 signalling [102].

Moreover, the study of variants of human MyD88 helped to document TLR2-MyD88 interaction. This is the case of the R753Q polymorphism in TLR2, which has been shown to impair MyD88 recruitment by the TLR2 receptor complex. This block, along with altering TLR6-TLR2 hetero-dimerization and TLR2 tyrosine phosphorylation, attenuates innate immune responses to mycobacteria [103]. A mutation in MyD88 with similar properties has also been reported for interactions with TLR4. In this case, the Asp299Gly polymorphism in human TLR4 interferes with the recruitment of MyD88 for the main signalling pathway and TRIF for the parallel pathway and alters the TLR4 signalling as a result [104]. A similar situation occurs with the Lys694Arg polymorphism of human TLR4 that leads to an attenuated response to LPS. In this case, the mechanism of action also relies on blocking the recruitment of MyD88 by TLR4 into the receptor complex [105].

### 6.3. Variability of TLR Adaptors Including MYD88 Across the Domestic Species

All TLRs (with the exception of TLR3) critically depend upon myeloid differentiation factor 88 (MyD88) to link bacterial recognition by TLRs with NF-B activation and cytokine production [75]. Evidence of the crucial role played by MyD88 as a signal transducer is provided by MyD88-knockout (MyD88^−/−^) mice, which die within 4 weeks from the time of infection with *M. tuberculosis* [41,75].

As documented by the studies in human MyD88, as summarised above, and by studies in model species, MyD88 is an important interactor of TLR molecules. However, the role of polymorphism of MyD88 has not been sufficiently studied until now in livestock species.

To date, only two publications have addressed the diversity of MyD88 in cattle. The work by Capparelli et al. from 2013 [106] concentrated on the effect on paratuberculosis (*Mycobacterium bovis* infection) in cattle. Although the effect of heterozygosity in the A625C polymorphic site has been detected, this work only covered a part of this gene using a limited set of polymorphisms.

On the other hand, the second foundational study on the bovine *MYD88* gene by Schaut et al. [107] showed that MyD88 deficiency reduced TLR4-mediated responsiveness to BVD virus type 2.

It can also be assumed that many polymorphisms in the conserved regions of the TLR adaptors escaped attention in view of the technical difficulties in the amplification, sequencing and genotyping of these sequences with standard laboratory techniques. This fact is due to an extremely high GC content, especially in the first half of the gene. In the case of bovine MYD88, the gene contains 64.8% GC in the first 1000 nucleotides and 69.0% in the first 500 nucleotides. A limited number of single-stranded stretches, as based on hybridisation prediction, can be used to design a series of amplification primers [Novák K., unpublished data]. The dimethylsulfoxide concentration and the Taq-polymerase brand have been optimised as well. The annealing temperatures allows to process the plate format or to use bulked PCR reactions to increase the throughput in the ongoing association study of the health traits of Czech Red Pied cattle.

On the other hand, the applied hybrid sequencing approach with HiSeq and PacBio technologies has also enabled identifying diversity in this bovine gene with a high level of reliability [Novák K., unpublished data].

In sheep and other small ruminants, screening for functionally relevant variations in MyD88 and other TIR-containing adaptors has started as well. The work by Arcangeli et al. [108] provided a survey of MyD88 variation within the range of sheep breeds reared in Italy, along with the survey of SNPs in other innate immune genes, like TMEM154 (transmembrane protein 154, functioning as a sensor of the electric field), TLR9, and CCR5. In addition, the association with resistance to infection by small ruminant lentiviruses (SRLVs) has been demonstrated.

Research on *MyD88* diversity appears to be more advanced in monogastric species, particularly in pigs [109]. The effect of MyD88 variants on the immune response upon the application of the Aujeszky vaccine against pseudorabies in piglets has been indicated in the association study [110]. In rabbits, mRNA analyses suggest that *MyD88* expression is associated with digestive disorders, although the effects of genetic variants have not yet been investigated [111].

Strong evidence linking polymorphisms in *MYD88* and functionally related genes to susceptibility to multiple infections in a local dog population was reported by Necesankova et al. [112]. These findings, obtained in an independent model species, confirm many of the conclusions drawn from studies in farm animals.

Highly promising results with respect to practical applications have been reported for the role of MyD88 in birds. A clear association has been demonstrated for SNPs located in the 3′-UTR of chicken MYD88 with respect to resistance to *Salmonella pullorum* infection [113].

A work performed in goose by Wei et al. [114] on the relation between TLR7, MyD88 and the response to the avian influenza virus H5N1 might mark the way for efficient breeding for resistance against this crucial infection in industrial chicken farming.

Surprisingly, a substantial amount of information on the variability in TIR-containing adaptors has been collected in fish species. The topic of TLRs and TIR-domain adaptors in this group of vertebrates has been addressed in detail with zebrafish as a representative species and the first-choice model in fish [115]. The expression of TLR and TIR domain adaptors in zebrafish have been summarised by Meijer et al. [116].

Research on TLR adaptors in fish, including economically important species, is represented by a study of *TLR9*, *MyD88*, and *TRAF6* expression in common carp [117]. It has been demonstrated that a limited translocation of MYD88 across the nuclear membrane, mediated by the ST2 protein atypically localised in rainbow trout, leads to quenching of TLR signalling [118].

### 6.4. Variability Affecting MyD88 Interactions with Other Members of the Toll Pathway

Not surprisingly, specific mutations in the Toll signalling pathway members can affect the interactions of key molecules on the molecular level, not the main functions performed by the individual members. Partly, the R753Q polymorphism in human TLR2 negatively affects TLR2 signalling, since it prevents TLR6-TLR2 hetero-dimerization. Consequently, subsequent TLR2 phosphorylation at tyrosine is affected as well and, in parallel, the MyD88 recruitment into the myddosome complexes [119]. It has been further shown that this polymorphism (R753Q) in Toll-like receptor 2 impairs MyD88 adaptor recruitment to TLR2. This leads to the attenuation of innate immune responses to mycobacteria [103]. This effect is not surprising in view of the specificity of TLR2 for recognition of bacterial lipoproteins [22].

A similar effect is observed for the polymorphism Asp299Gly in human TLR4, which affects interactions with TLR6 and MyD88 [104]. Likewise, the formation of TLR4-MyD88 complexes by recruitment of MyD88 is prevented by the Lys694Arg polymorphism, resulting in blunted responses to bacterial LPS [34].

### 6.5. Importance of the Haplotype Structure

It is well documented that synonymous mutations or even mutations in the noncoding regions can affect phenotypic traits in association studies. This can be exemplified by the reported effect of 1313G>A in *TLR2* on the susceptibility to PTB [62]. However, this SNP is a part of a haplotype, i.e., a variant block in cis, that increases susceptibility to PTB as a whole.

Consequently, the effects of haplotypes should be considered in place of the effects of the causal SNPs alone in association studies. In contrast to simple SNPs, haplotypes comprising particular blocks of SNPs and identified via tagSNPs integrate causal polymorphisms in the coding and regulatory regions [120]. Therefore, knowledge of haplotype structure is a prerequisite for objective association studies with consequences for breeding.

The switch towards the use of the haplotype structure of the whole genome or at least the particular genes of interest is perceived in the area of livestock genomics, in parallel to the leading human genomics [121]. In the livestock example of cattle, the first haplotype-resolved genomes were obtained for individual animals from the populations of Brahman and Angus breeds [122]. In spite of the considerable labour and expense involved in the characterisation of the haplotype structure across the whole studied population, reconstruction of the haplotype structure on a smaller scale—focusing on a specific set of studied genes—can be useful in performing the association studies of the immune genes, e.g., [63]. When haplotypes are used to detect associations, the results are not affected by errors caused by the linkage of a marker SNP with other polymorphisms, including the causal variants underlying the traits studied. The differentiation of the genetic factors studied on the haplotype level provides results free of this error, otherwise very common in population studies based on genotyping arrays, usually built on common and arbitrarily selected diagnostic markers [123]. On the other hand, considering the real haplotype structure leads to an increase in the number of genotypic classes associated with a drop in the abundance of minor classes. This is contradictory to the resolution power of the used statistical procedures, such REML [124], and might prevent the interpretation of the results of the otherwise costly association study.

An example of this approach to proving the suspected effect of variations in the members of the Toll signalling pathway has been provided by Mollaki et al. [125]. The haplotype blocks of variants in *TLR9* and *MYD88* have been shown to be associated with the development of Hodgkin’s lymphoma in humans.

A recent interpretation of haplotypes in bovine *TLR*s in parallel to these genes of a rodent model bank vole (*Myodes glareolus*) should help to identify the functional reasons for a stable disequilibrium in the haplotypes in these genes [126].

## 7. Prospect for Using Toll Pathway Variability for Increasing the Efficiency of Breeding in Livestock Species

Although the efforts to apply diversity in immune genes to livestock breeding have a long tradition [3], the situation in immune gene variation knowledge in livestock species is extremely favourable for the utilisation of this opportunity in breeding for health traits. However, with the ongoing characterisation of livestock genomes—partly through projects such as FAANG (Functional Annotation of Animal Genomes, https://faang.animalgenome.org/, accessed on 5 May 2026) and the recent BovReg (https://bovreg.eu/, accessed on 5 May 2026), which focus on understanding cattle genomes—the importance of variability in regulatory, noncoding regions has become increasingly evident. As a result, the data collected in these databases on noncoding region variability now offer new opportunities to harness natural genetic diversity for improving the animal immune system.

Despite the wealth of data from TLR structure and function modelling and from in vitro studies, predicted effects must be validated on the organismal level. The uniform gene pool and uniform conditions of husbandry in main livestock breeds facilitate the detection of genetic effects at the population level.

That is why performing so-called association studies is the next logical step after the documentation of polymorphism in the genes of interest in the particular population of a livestock or a model species.

For association studies in livestock species, it is convenient to use the economic or veterinary estimates of animals obtained over the course of standard husbandry on a large scale and systematically collected and archived by breeders’ associations. Collecting the same amount of data through individual research projects would not be realistic in most cases.

It can be assumed that association studies investigating the effects of variants in TLR genes—generally in genes involved in the Toll signalling pathway and innate immunity—in livestock species parallel the numerous studies conducted in biomedical research.

Targeted genotyping of variations in causal genes for health traits can be considered a complement to the generally adopted genomic selection in cattle [123]. Its use might lead to faster improvements in the population structure for the desired gene variants. Accordingly, breeding for health traits in cattle is an alternative to veterinary measures [127].

The study by Capparelli et al. [106], which is unique to date, demonstrated that heterozygosity at the MyD88 A625C polymorphic site is associated with resistance against active—but not latent—*M*. *bovis* infection in cattle.

Polymorphism in the *MYD88* gene has recently been associated with resistance to general stress in humans [128]. At the same time, a reduction in the susceptibility of animals to stress factors is one of the current directions in livestock breeding. Therefore, the described polymorphism of this key gene in the CRP cattle population [Novák K., unpublished data] might find application in resilience breeding in the future.

## 8. Conclusions

Undoubtedly, the future translational processes anticipated will contribute to animal breeding to a greater extent than has been achieved so far. Although a sufficient amount of knowledge has been accumulated on Toll-like receptor diversity, the attention paid to other members of the pathway is not adequate for their importance and expected variability. More intense study of the initial members of the pathway sharing highly conserved TIR regions, like MyD88, might bring about solutions to the actual challenges in breeding for infection resistance. The highly conserved and evolutionarily optimised structure of TIR regions in Toll signalling adaptors means that any structural modification can lead to pronounced changes in the activity and specificity of the innate immune response. Consequently, this research direction in livestock species has the potential to yield significant clinical and breeding applications.

This research is also synergistic with the recent progressive approaches in the field of animal molecular genetics and genomics. It can be expected that the ongoing characterisation of the genomes of major livestock species at the haplotype resolution, as well as advanced methods of large-scale resequencing, will enable more precise estimates of available diversity. Extensive use of new techniques should also accelerate the previously delayed research of the highly conserved structures of the Toll signalling pathway adaptors in livestock species.

## Data Availability

No new data were created during the study.

## Abbreviations

The following abbreviations are used in this manuscript:

ANOVA	one-factor analysis of variance
APEX1	apurinic/apyrimidinic endonuclease 1
CDS	coding DNA Sequence
CpG	5′-C-phosphate-G-3′
DC	dendritic cell
DNA	deoxyribonucleic acid
EBI	European Bioinformatics Institute, Cambridge, VB
EDTA	ethylenediaminetetraacetic acid
gDNA	genomic deoxyribonucleic acid
G^−^, G^+^	Gram-negative and -positive bacteria, respectively
IFN-α	type I interferon-α
IKK	inhibitor of κB Kinase
IKK-γ	inhibitor of NF-κB kinase regulatory subunit γ
IL-1R	interleukine-1 receptor
IRAKs	interleukin-1 receptor (IL-1R) associated kinases
IRF-7	interferon regulatory factor 7
ITAM	immunoreceptor tyrosine-based activation motif
LPS	lipopolysaccharide
LRR	leucine-rich repeats
Mal/TIRAP	TIR-domain-containing adaptor/MyD88 adaptor-like
MD-2	myeloid differentiation factor 2
MyD88	myeloid differentiation primary response gene 88
NK	natural killer cells
NKT	natural killer T cells
NCBI	National Center for Biotechnology Information
NF-κB	nuclear factor κB
NGS	next-generation sequencing
NOX2	NADPH oxidase 2
PAMPs	pathogen-associated molecular patterns
PCR	polymerase chain reaction
PLCγ2	phospolipase C gamma 2
PRR	pattern recognition receptors
RNA	ribonucleic acid
SNP	single nucleotide polymorphism
SRLVs	small ruminant lentiviruses
SYK	spleen tyrosine kinase
TAB	TAK-1-binding proteins
TAK1	TGF (transforming growth factor)-β-activated kinase 1
TBL1XR1	transducin beta-like 1 receptor 1
TIR	Toll/IL-1R/R gene region
TLR	Toll-like receptors
TMEM154	transmembrane protein 154
TNF	tumour necrosis factor
TRAF3	TNF receptor associated factor 3
TRAF6	TNF receptor associated factor 6
